# Comparative Appraisal of Biomass Production, Remediation, and Bioenergy Generation Potential of Microalgae in Dairy Wastewater

**DOI:** 10.3389/fmicb.2019.00678

**Published:** 2019-03-29

**Authors:** Amandeep Brar, Manish Kumar, Nidhi Pareek

**Affiliations:** Biocatalysis and Bioprocess Laboratory, Department of Microbiology, School of Life Sciences, Central University of Rajasthan, Ajmer, India

**Keywords:** dairy wastewater, microalgae, phycoremediation, bioenergy, environment

## Abstract

The present study is a trail to integrate the phycoremediation and bioenergy production from microalgal species cultivated in the dairy wastewater (DWW). Higher biomass productivities for *Chlorella pyrenoidosa* (24.44 ± 8.02 mg L^-1^d^-1^), *Anabaena ambigua* (23.64 ± 5.69 mg L^-1^d^-1^) and *Scenedesmus abundans* (18.72 ± 2.06 mg L^-1^d^-1^) were recorded in 3:1 DWW over the control. The microalgal species have effectively reduced the BOD by 56%, COD by 77%, nitrate by 88%, and phosphate by 85% following 25 days of the cultivation in the 3:1 DWW. The total lipid content was 10.36, 13.13, and 16.93% of dry matter of biomass in *C. pyrenoidosa, A. ambigua*, and *S. abundans*, respectively following 25 days of cultivation in the 3:1 ratio of DWW. The biochemical characterization revealed that the protein content was 21.8% in *C. pyrenoidosa*, 17.73% in *A. ambigua* and 34.06% in *S. abundans*. The estimation of theoretical methane potential suggested that the microalgal species have the desirable possibility of biogas generation. The results have marked the achievability of an integrated process for the remediation and bioenergy production by the employment of microalgal species.

## Introduction

Efforts for the development of sustainable energy sources are under fierce global development as the petroleum reserves are finite and have reached a pinnacle. The extent of industrialization and the pressure exerted by the ever-growing population has resulted in the release of a gigantic amount of wastewaters which are the by-products of various agricultural, commercial, domestic and industrial activities into the environment. The dairy industry is not only one of the most polluting industries in terms of the effluent volume generated but also, in terms of the nature of the effluent. The annual production capacity of Indian dairy industries is approximately 5.5 × 10.66 Kl. It is estimated that 8.7 cubic meters of water per kiloliter of milk produced are consumed to the generation of 6.0 cubic meter of water waste (Annual report, central pollution control board). The wastewater generated includes the production line cooling water, domestic wastewater, acid whey and sweet whey which lead to the change in the quality and quantity of wastewater. It is reported that the sweet whey is the most polluting effluent due to its biochemical composition, i.e., rich in organic matter (lactose, nitrates, phosphorous, protein etc.) (Shete and Shinkar, 2013). The wastewater contains high amounts of nitrogen and phosphorous that poses a serious concern to the environment in the form of eutrophication and the research has been focused on the removal of nutrients from the wastewater before its discharge into the natural water bodies (Monfet and Unc, 2016). These nutrients are usually removed in the tertiary phase of treatment by employing biological process in form of anaerobic digestion, usually followed by nitrification and denitrification. The several rounds of these coupled processes are a prerequisite to achieve the permissible limits (Queiroz et al., 2007). The methodologies that are currently applied to the removal of these nutrients requires several tanks and internal recycling of activated sludge leading to the complexity and increased cost and demands high energy input.

Consequently, the use of microalgae has been proposed as a crucial platform for renewable energy production that will not compete with the agricultural resources and can remediate the wastewater as requires a large amount of nitrogen and phosphorous for its growth (Goncalves et al., 2017). Moreover, high nitrate and phosphate removal efficiency of different microalgal species have already been reported by many researchers (Li et al., 2011; Sydney et al., 2011). Microalgal cultivation in wastewater has gained enormous popularity due to its efficiency for the production of biomass at reduced costs and lower environmental impacts as nutrients and freshwater are not a requisite. They have the capability to capture carbon at a faster rate in comparison to terrestrial crops and can accumulate high percentage of carbohydrates, lipids or proteins in their biomass over a short period of time (Hernández et al., 2014). These products can be easily processed into desirable biofuel viz. biodiesel, bioethanol or biogas (Brennan and Owende, 2010). The coupling of wastewater remediation with microalgae cultivation is considered as a sustainable process as it does not lead to secondary pollution due to reutilization of produced biomass in bioenergy production.

Currently, commercially available biofuels are largely produced from the edible food crops (viz. corn starch, sugarcane, soybean, or oilseed rape) but there is a discord regarding the economical competitive behavior of crop-based biofuels over the fossil fuels and their impact on the food security (Pittman et al., 2011). So, the biofuels that are derived from the microalgae have been favored and proposed as an alternative approach without any negative effect on the agriculture system.

It is evident from the above discussion that the coupling of phycoremediation of dairy wastewater (DWW) followed by bioenergy production from the resulting biomass holds a great promise for the sustainable development and environment waste management. To the best of our knowledge *C. pyrenoidosa, A. ambigua*, and *S. abundans* have not been previously employed for the remediation of DWW under similar conditions. Their growth kinetics were studied at different ratios of DWW and the best ratio was further selected for the remediation. The biomass produced was further investigated for its carbohydrate, protein and lipid content so as to determine its potentiality for production of biofuels.

## Materials and Methods

### Materials

Dairy wastewater (DWW) was collected from Saras dairy plant, Jaipur (26° 86′ N, 75° 81′ E), Rajasthan, India and was stored at 4°C the further analysis. Saras dairy plant has a processing capacity of 500 thousand liters per day and its aseptic packaging station processes 100 thousand liters per day. The initial pH of DWW collected was 4.34 (Eutech pH 150).

### Characterization of DWW

The characteristics of the dairy plant effluent largely depend on the quantity of milk processed and the nature of the manufactured product. The DWW was white in color and the fermentation of milk sugar to lactic acid imparts its acidic pH. The decomposition of casein leads to the formation of black sludge and the presence of butyric acid might be responsible for foul smell. The DWW was initially characterized for the pH, biological oxygen demand (BOD), chemical oxygen demand (COD), total organic carbon, total dissolved carbon, total nitrogen, total organic carbon and total organic phosphate as summarized in Table 1, following the APHA protocols (American Public Health Association [APHA] et al., 1998).

**Table 1:** Characterization of dairy waste water.

Parameters	Value
pH	4.34 ± 0.5
Biological oxygen demand (BOD) (mgl^-1^)	245.95 ± 8.48
Chemical oxygen demand (COD) (mgl^-1^)	1280 ± 226.47
Total organic carbon (TOC) (mgl^-1^)	1372 ± 150
Dissolved organic carbon (DOC) (mgl^-1^)	890 ± 52
Dissolved organic nitrogen (DON) (mgl^-1^)	266 ± 11
Total nitrogen (TN) (mgl^-1^)	363.97 ± 23.93
Total phosphate (ppm)	19583 ± 424
Nitrate (mgl^-1^)	249.94 ± 12.72

*All the parameters were analyzed as per the protocols mentioned in American Public Health Association [APHA] et al. (1998). Values represent the mean ± SD of three replicates.*

### Microorganisms and Growth Conditions

The cyanobacterium *Anabaena ambigua* (NCIM No. 2785), the microalga *Chlorella pyrenoidosa* (NCIM No. 2738), and *Scenedesmus abundans* (NCIM No. 2897), were procured from National Collection of Industrial Microorganism (NCIM), Pune, Maharashtra, India. They were maintained as per their recommendations in Fog’s growth medium. For the growth of *C. pyrenoidosa*, the Fog’s medium was supplemented with an additional 2% KNO_3_. The composition of Fog’s growth medium as described by NCIM, Pune includes MgSO_4_.7H_2_O (0.2 g l^-1^), K_2_HPO_4_ (0.2 g l^-1^), CaCl_2_.2H_2_O (0.1 g l^-1^), Fe-EDTA solution (5 ml l^-1^) and micronutrients H_3_BO_3_ (286 mg l^-1^), MnCl_2_.4H_2_O (181.0 mg l^-1^), ZnSO_4_.7H_2_O (22 mg l^-1^), Na_2_MoO_4_.2H_2_O (39 mg l^-1^), CuSO_4_.5H_2_O (8 mg l^-1^). The pH of 7.2 was maintained throughout the growth period. The batch cultures were provided with the white fluorescent cool light (4^∗^40 watt) and the temperature was maintained at 25 ± 2°C with a photoperiod of 16:8 h. The flasks were manually shaken (four to five times a day) to prevent the attachment of algal cells to the culture flasks.

### Growth of Microalgae in DWW

The growth profile of different species was studied by the dilution of wastewater into different ratios with BG-11 medium. Volume ratios of DWW with BG-11 employed in the present study were 1:3 DWW (1 part of DWW and 3 parts of BG-11 medium), 1:1 DWW (equal parts of DWW and BG-11 medium), 3:1 DWW (3 parts of DWW and 1 part of BG-11 medium) and 1:0 DWW (only DWW). The composition of BG-11 medium includes NaNO_3_ (1.5 g l^-1^), K_2_HPO_4_ (0.04 g l^-1^), MgSO_4_.7H_2_O (0.075 g l^-1^), CaCl_2_.2H_2_O (0.036 g l^-1^) citric acid (0.006 g l^-1^), ferric ammonium citrate (0.006 g l^-1^), EDTA (disodium salt) (0.001 g l^-1^), Na_2_CO_3_ (0.02 g l^-1^), and 1 ml of trace element solution containing micronutrients in the form of H_3_BO_3_ (2.86 g l^-1^), MnCl_2_.4H_2_O (1.81 g l^-1^), ZnSO_4_.7H_2_O (0.22 g l^-1^), Na_2_MoO_4_.2H_2_O (0.39 g l^-1^), CuSO_4_.5H_2_O (0.079 g l^-1^), Co(NO_3_)_2_ (0.05 g l^-1^) (Stanier et al., 1971). The growth of *A. ambigua, C. pyrenoidosa* and *S. abundans* at various ratios of autoclaved DWW was compared with the growth in the control (0:1 DWW; BG-11 medium only). The pH was adjusted to 6.5 by using 0.1M NaOH buffer. The inoculum (10%, v/v) used was homogenous algal suspension having absorbance of 2.0 at 680 nm. The growth was measured every 24th h in terms of total chlorophyll content using methanol as a solvent and 90% methanol was used as a control. The absorbance was measured at 665 and 750 nm (Spectronic 200-Thermo Scientific, Germany) by the method of Marker et al. (1980). The specific growth rate (day^-1^) was calculated according to equation:

(1) $$\mu ({\text{day}}^{\text{-1}})\;\text{=}\;\text{ln}({\text{N}}_{\text{2}}{\text{/N}}_{\text{1}})({\text{t}}_{\text{2}}{\text{-t}}_{\text{1}})$$

where, N_1_ and N_2_ represent growth of algae (μg m l^-1^) at time t_1_ and t_2_ (Issarapayup et al., 2009). The biomass productivity (μg m L^-1^d^-1^) was calculated by the equation:

(2) $$\text{P}\;\text{=}\;({\text{N}}_{\text{i}}{\text{-N}}_{\text{o}})\text{/}({\text{t}}_{\text{i}}{\text{-t}}_{\text{o}})$$

where, N_i_ and N_o_ are the biomass (μg m l^-1^) at time t_i_ and t_o_ respectively (Griffiths et al., 2014). The ratio of DWW: BG-11 that resulted into the maximum growth was selected for further studies.

### Phycoremediation of DWW

The experiment was carried in Erlenmeyer flasks (1 L) under batch conditions with a working volume of 600 ml for 25 days using the 3:1 ratio of DWW (diluted with BG-11 medium). The optimum pH of 6.5 and a temperature of 25 ± 2°C were maintained throughout the experiment with an illumination intensity of 4 tube-lights (40 watts each) for 16:8 h. Autoclaved 3:1 ratio of DWW (3 parts of DWW and 1 part BG-11 medium) without algal inoculation was used as a control. Every 5th day, aliquots were withdrawn and analyzed for BOD (titrimetric method), COD (closed reflux, dichromate titrimetric method), nitrate (spectrophotometric method) and phosphate (ascorbic acid method). These estimations for the study of relative remediation potential of microalgal strains were performed in triplicates following the standard protocols as mentioned in the American Public Health Association [APHA] et al. (1998). Removal efficiency (RE, %) of the strains used was calculated by the equation:

(3) $$\text{RE}(\%)\;\text{=}\;[({\text{S}}_{\text{0}}{\text{-S}}_{\text{f}}){\text{/S}}_{\text{0}}]\;\times \;\text{100}$$

where, S_0_ and S_f_ are the nutrient concentrations at the beginning and the end of the experiment, respectively. The removal rate (RR, mg l^-1^ d^-1^) of the analyzed nutrients was calculated using the equation:

(4) $$\text{RR}\;\text{=}\;({\text{S}}_{\text{0}}{\text{-S}}_{\text{i}})\text{/}({\text{t}}_{\text{i}}{\text{--t}}_{\text{0}})$$

Where, S_i_ is the nutrient concentration at time t_i_.

### Determination of Carbohydrate, Lipid, Protein, and Total Kjeldahl Nitrogen Content

The harvested algal biomass following 25 days of growth in 3:1 ratio of DWW was dried in the oven at 60°C and was analyzed for total carbon, nitrogen and hydrogen by a CHNS/O analyzer (Thermo Scientific Flash 2000 CHNS/O elemental analyzer). Briefly, powdered biomass (100 mg) was hydrolyzed with 2.5 N HCl in the boiling water bath (3 h) followed by neutralization with sodium carbonate. An aliquot of 0.1 ml was diluted to 1 ml in a clean test tube. The carbohydrate estimation was performed by the phenol-sulfuric acid method (Dubois et al., 1956) employing glucose as the standard. Briefly, carbohydrates (polysaccharides) are hydrolyzed into simple sugars (monosaccharides) employing sulfuric acid that led to the formation of furfural and hydroxyl furfural, which further forms an aromatic complex with phenol and yields a change in the color.

Total lipid content was estimated according to the optimized calorimetric sulfo-phospho-vanillin method (SPV method) by Chabrol and Charonnat (1937) and Klin et al. (2018) during both remediation studies and biochemical characterization of biomass. For the extraction of lipids, algal cultures (1 ml, obtained during remediation after 5, 15, and 25 days of incubation) were centrifuged (10,000 rpm, 5 min) and re-suspended in methanol (0.5 ml). For the effective extraction of lipids, the samples were vigorously shaken with glass beads followed by the addition of chloroform (1 ml) (Folch et al., 1957). The samples were centrifuged to collect the supernatant with further addition of NaCl (0.8%, 0.2 ml). The samples were left at room temperature for the formation of two separate layers. The upper layer was discarded and the lower layer was dried at 50°C. Then, 0.3 ml of concentrated sulfuric acid was added and was heated at 90°C for 10 min. One milliliter of sulfo-phospho-vanillin (SPV) reagent was added to fully stain the fatty acids. The absorbance of the final solution was measured at 525 nm (Knight et al., 1972). Cholesterol was used as a standard for lipid estimation. Additionally, for determination of total lipid content (biochemical characterization) the biomass obtained (following 25 days of incubation) was washed with distilled water and dried in an oven. Biomass (100 mg) was then hydrolyzed with hydrochloride in boiling water bath (3 h) followed by lipid quantification employing the aforementioned SPV method.

The total Kjeldahl nitrogen was estimated using the standard protocol mentioned in the APHA [IS-3025, part-3025 (part 34)]. For the determination of total protein content of dried algal biomass harvested after 25 days of growth, the total nitrogen was analyzed by CHNO analyzer (Thermo Scientific Flash 2000 CHNS/O elemental analyzer). Protein content has been estimated based on the total nitrogen content as nitrogen content is multiplied by a factor to arrive at protein content. It is based on assumption that carbohydrate and fat do not contain nitrogen and nearly all the nitrogen is present as proteins. On this basis, the average nitrogen content was found to be 16%, which led to the calculation N × 6.25 (1/0.16 = 6.25) (Becker, 1994; Pancha et al., 2014) and the protein content was estimated by the following equation:

(5) Crude protein (%) = total nitrogen (%) × 6.25

### Theoretical Methane Potential

Based on the atomic composition obtained by the CHNO analyzer for the dried algal biomass, the stoichiometric equation was used to calculate the theoretical methane potential (BMP_th_) by Boyle’s equation. Boyle’s equation considers the presence of proteins and ammonia.

(6) $$\begin{array}{c} C_{n}H_{a}O_{b}N_{c}+(n-a/4-b/2+3c/4)H_{2}O\to \;(n/2+a/8\\ -b/4-3c/8)CH_{4}+(n/2-a/8+b/4+3c/8)CO_{2}+cNH_{3} \end{array}$$

(7) $${\text{BMP}}_{\text{th}}=\frac{22.4\left(\frac{n}{2}+\frac{a}{8}-\frac{b}{4}-\frac{3c}{8}\right)}{12n+a+16b-14c}$$

The equation determines the methane productivity of a specific substrate from its elemental composition. This equation does not distinguish between the biodegradable and non-biodegradable organic matter and the matter that will be utilized by the microorganisms for its growth will not contribute to the theoretical biochemical methane potential (Lesteur et al., 2010).

## Results and Discussion

### Growth of Microalgae in DWW

**FIGURE 1:**
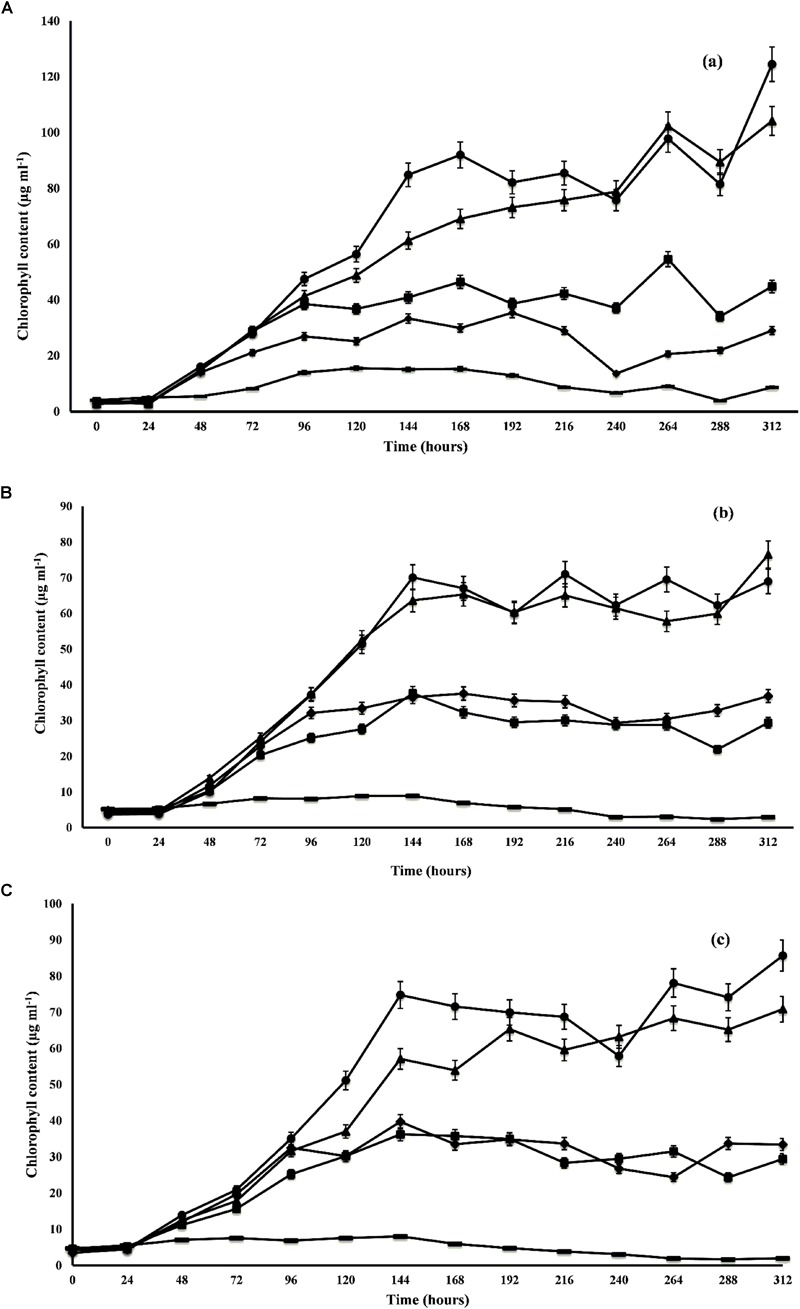
Growth profile of *C. pyrenoidosa* **(A)**, *S. abundans* **(B)**, and *A. ambigua* **(C)** at different ratios of DWW and standard growth medium (control; ■ 1:3 DWW; ▲ 1:1 DWW; ● 3:1DWW; ◆ 1:0 DWW) (Values represent the mean ± SD of three replicates).

The growth profile of microalgal species was studied at different ratios of DWW for 14 days. The maximum growth in terms of total chlorophyll content was observed by *C. pyrenoidosa* (104.16 ± 0.59 μg m l^-1^) at the 14th day employing 1:1 ratio of DWW (equal parts of DWW and BG-11 medium). In the 3:1 ratio of DWW (3 parts of DWW and 1 part BG-11 medium), maximum chlorophyll content shown by *C. pyrenoidosa* at 12th day was 97.83 ± 6.72 μg m l^-1^ followed by *A. ambigua* (85.65 ± 4.24 μg m l^-1^ at 7th day) and *S. abundans* (70.09 ± 0.88 μg m l^-1^ at 7th day) (Figure 1). However, it is worth noting that the highest chlorophyll content recorded in control experiment was 35.42 ± 0.55 μg m l^-1^ for *C. pyrenoidosa*, 8.93 ± 0.19 μg m l^-1^ for *S. abundans* at 6th day and 8.02 ± 0.15 μg m l^-1^ for *A. ambigua* at the 7th day of the experiment. For all the microalgal species, significantly higher growth was recorded in the 3:1 ratio of DWW over all other ratios and the control (*p* < 0.0001). The batch culture of the microalgal species used exhibited all the characteristic phases of a growth curve for all the ratios of DWW employed in the study. *C. pyrenoidosa* remained in the exponential phase for 168 h of growth (Figure 1A) whereas *A. ambigua* and *S. abundans* remained in the log phase for 144 h of growth for all the ratios of DWW (Figure 1B,C). The growth kinetics was studied in terms of biomass productivity and specific growth rates for all the microalgal species at all the ratios. The maximum biomass productivity recorded for *C. pyrenoidosa* was 42.99 ± 1.09 mg l^-1^ d^-1^ at 13th day whereas it was 18.72 ± 2.06 mg l^-1^ d^-1^ for *S. abundans* and 23.65 ± 2.29 mg l^-1^ d^-1^ for *A. ambigua* at the 6th day of the experiment in the 3:1 ratio of DWW. The control showed highest biomass productivity of 4.76 ± 0.77 mg l^-1^ d^-1^ for *C. pyrenoidosa*, 0.58 ± 0.134 mg l^-1^ d^-1^ for *S. abundans* and 0.48 ± 0.03 mg l^-1^ d^-1^ for *A. ambigua* at 13th day of the experiment. For all the microalgal species studied, the highest specific growth rate was observed at the 2nd day of the experiment, i.e., 1.32 ± 0.08 d^-1^ for *C. pyrenoidosa*, 0.98 ± 0.10 d^-1^ for *S. abundans* and 1.12 ± 0.04 d^-1^ for *A. ambigua* for 3:1 ratio of DWW. Moreover, for the 1:1 ratio of DWW, the maximum specific growth rate was recorded at 2nd day of the experiment. In the control, *C. pyrenoidosa* showed significantly higher specific growth rate (0.79 ± 0.12 d^-1^ on the 13th day) over the other two species at all the intervals of the time period. In 1:0 ratio of DWW, the biomass productivity and specific growth rate both were lower than that 3:1 and 1:1 ratio of DWW. Although the microalgal species showed notable growth in both 3:1 and 1:1 ratio of DWW, however, in lieu of the process economics of the remediation process, 3:1 ratio was selected for the further experiments (Table 2).

**Table 2:** Growth profile of *A. ambigua, C. pyrenoidosa*, and *S. abundans* in dairy wastewater in terms of specific growth rate and biomass productivity.

Species	*A. ambigua*									
**Parameter μ (d^-1^)**		**P(mg L^-1^ d^-1^)**								

**Treatment**	**Control**	**1:3 DWW**	**1:1 DWW**	**3:1 DWW**	**1:0 DWW**	**Control**	**1:3 DWW**	**1:1 DWW**	**3:1 DWW**	**1:0 DWW**
2nd day	0.251 ± 0.01	0.761 ± 0.08	1.001 ± 0.05	1.119 ± 0.04	0.998 ± 0.003	1.564 ± 0.03	5.958 ± 1.06	7.866 ± 0.11	9.351 ± 0.32	7.626 ± 0.05
4th day	0.092 ± 0.002	0.479 ± 0.001	0.574 ± 0.03	0.521 ± 0.03	0.502 ± 0.01	0.672 ± 0.04	9.581 ± 0.16	13.81 ± 0.66	14.17 ± 1.94	12.82 ± 0.35
6th day	0.061 ± 0.01	0.179 ± 0.14	0.429 ± 0.18	0.379 ± 0.18	0.273 ± 0.08	0.464 ± 0.08	6.005 ± 4.77	20.08 ± 9.87	23.64 ± 5.69	9.508 ± 3.01
8th day	0.227 ± 0.04	0.023 ± 0.04	0.192 ± 0.001	0.023 ± 0.001	0.038 ± 0.01	0.906 ± 0.25	0.813 ± 0.15	11.38 ± 0.05	11.63 ± 0.17	1.314 ± 0.44
10th day	0.178 ± 0.05	0.038 ± 0.06	0.069 ± 0.02	0.321 ± 0.21	0.085 ± 0.01	1.183 ± 0.19	1.125 ± 0.20	3.586 ± 1.25	10.84 ± 1.89	6.881 ± 2.65

**Species**	***C. pyrenoidosa***								

2nd day	0.085 ± 0.01	1.694 ± 0.11	1.549 ± 0.11	1.321 ± 0.08	1.464 ± 0.18	0.459 ± 0.84	12.01 ± 0.51	11.65 ± 0.78	11.74 ± 0.69	10.73 ± 0.65
4th day	0.533 ± 0.14	0.291 ± 0.04	0.354 ± 0.15	0.530 ± 0.07	0.253 ± 0.18	5.765 ± 1.45	9.716 ± 1.35	12.26 ± 5.01	19.59 ± 3.83	5.838 ± 0.83
6th day	0.107 ± 0.04	0.107 ± 0.02	0.228 ± 0.002	0.406 ± 0.09	0.282 ± 0.02	0.423 ± 0.66	4.128 ± 0.47	12.51 ± 0.35	28.44 ± 8.02	8.194 ± 0.44
8th day	-0.17 ± 0.00	0.085 ± 0.002	0.057 ± 0.01	0.037 ± 0.08	0.104 ± 0.05	4.185 ± 0.12	3.649 ± 0.35	4.128 ± 0.42	9.841 ± 0.62	5.463 ± 0.17
10th day	-0.26 ± 0.03	0.387 ± 0.12	0.254 ± 0.003	0.127 ± 0.04	0.407 ± 0.24	2.335 ± 0.34	5.213 ± 0.43	3.002 ± 0.35	22.02 ± 4.36	6.985 ± 0.54

**Species**	***S. abundans***								

2nd day	0.222 ± 0.12	0.717 ± 0.004	1.103 ± 0.22	0.975 ± 0.10	1.036 ± 0.16	1.329 ± 0.71	5.249 ± 0.08	9.237 ± 1.42	6.249 ± 0.73	7.615 ± 1.71
4th day	0.097 ± 0.01	0.222 ± 0.12	0.393 ± 0.01	0.439 ± 0.02	0.341 ± 0.09	0.146 ± 0.08	4.879 ± 2.41	12.16 ± 0.38	13.22 ± 0.35	9.299 ± 2.65
6th day	0.004 ± 0.03	0.304 ± 0.22	0.186 ± 0.01	0.311 ± 0.03	0.089 ± 0.07	0.036 ± 0.03	10.03 ± 2.71	11.05 ± 7.01	18.72 ± 2.06	3.086 ± 2.53
8th day	0.177 ± 0.06	0.019 ± 0.02	0.077 ± 0.04	0.167 ± 0.02	0.010 ± 0.001	0.657 ± 0.38	0.542 ± 0.06	4.712 ± 3.65	10.93 ± 1.35	0.375 ± 0.05
10th day	0.025 ± 0.17	0.042 ± 0.005	0.036 ± 0.02	0.109 ± 0.06	0.029 ± 0.01	0.073 ± 0.01	0.042 ± 0.01	3.669 ± 1.53	7.172 ± 4.36	1.105 ± 0.31

*μ, Specific growth rate; P, Biomass productivity. Values represent the mean ± SD of three replicates.*

The above results were in agreement with the earlier reported observations that the higher concentration of nutrients favors the microalgal growth resulting in higher growth potential (Prajapati et al., 2013). Wang et al. (2010b) compared the growth of *Chlorella* sp. in the local municipal wastewater and concluded that the wastewater centrate generated from the sludge exhibited a significant increase in the growth due to its higher nitrogen, phosphate and COD values. The pattern of growth proves that the wastewater can provide ample nutrients in the form of nitrate and phosphate for the autotrophic growth and the COD for the heterotrophic growth under dark conditions. The increase in growth and decrease in the COD levels simultaneously depicts the ability of microalgal species to grow under dark conditions (Abou-Shanab et al., 2011).

### Phycoremediation of DWW

Phycoremediation was studied at 3:1 ratio of DWW, selected on the basis of growth kinetics of the microalgal species. This ratio has supported the remarkably higher growth of microalgal species as compared to the BG-11 medium (control for growth experiment). The control used in the phycoremediation experiment was autoclaved 3:1 ratio of DWW (without any inoculation of microalgal species). The objective of using the autoclaved 3:1 DWW was to minimize the growth of native microorganisms and to study the effect of inoculated species only, as native microorganisms can compete with the inoculated species for nutrition and space. Moreover, abundant literature has been available supporting the fact that bacteria and fungi are effective agents of remediation. So, the use of autoclaved DWW has curtailed the role of native microorganisms in the remediation. The phycoremediation efficiency was studied in terms of reductions in the BOD, COD, nitrate and phosphate contents.

#### BOD

**FIGURE 2:**
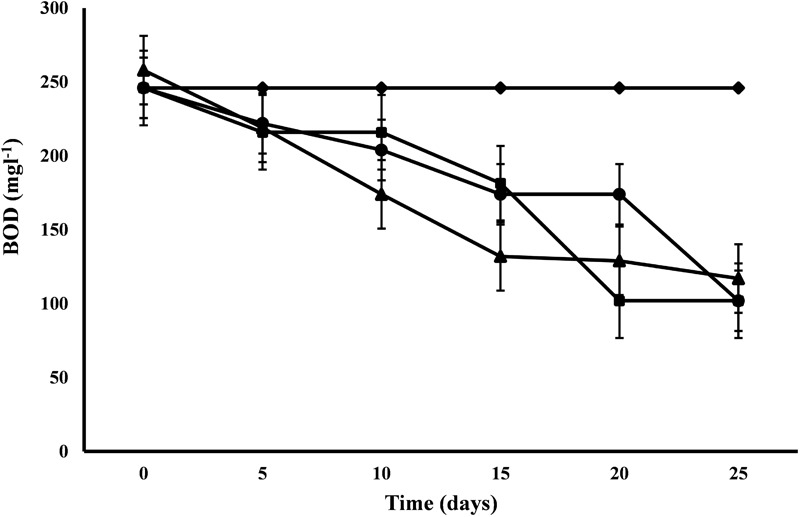
BOD reduction at alternate of 5 days in 3:1 ratio of DWW treated with *A. ambigua, C. pyrenoidosa* and *S. abundans* (◆ -control; ■ -*C. pyrenoidosa*; ▲ -*S. abundans*; ● -*A. ambigua*) (Values represent the mean ± SD of three replicates).

*C. pyrenoidosa* and *A. ambigua* were at par with each other for BOD removal efficiency (58.54%) following 25 days of phycoremediation experiment, while 52.44% BOD removal efficiency was observed for *S. abundans*. *S. abundans* showed a gradual reduction in BOD levels till 15th day but after that there was no visible reduction (Figure 2). The BOD in control was 245.95 mg l^-1^ and it was reduced to 101.98 mg l^-1^ in both *C. pyrenoidosa* and *A. ambigua* whereas it was 116.98 mg l^-1^ in *S. abundans*. Maximum removal rate (7.59 mg L^-1^d^-1^) was observed for *S. abundans* after the 15th day of the experiment (Table 3). In an experiment, the *Nostoc* sp. cultivated in the dairy effluent has reduced the BOD levels by 40.25%. The reduction in the BOD levels might be attributed to the removal of dissolved organic compounds and derivatives to a notable extent during the treatment process (Kotteswari et al., 2012). Kotteswari et al. (2007) have reported 47.34% BOD reduction in the dairy effluent by the treatment of *Spirulina platensis*. Hena et al. (2015) has reported BOD reduction up to 82% by using microalgal consortium for the remediation of dairy farm wastewater. Similar results (82% reduction) were also reported by Ummalyma and Sukumaran (2014) after 15 days of incubation of microalgal species in the dairy effluent.

**Table 3:** Reduction efficiency (RE) and removal rate (RR) of *A. ambigua, C. pyrenoidosa*, and *S. abundans* in 3:1 ratio of DWW.

	*A. ambigua*			*C. pyrenoidosa*			*S. abundans*		
Parameters	RE (%)	RR (mgl^-1^d^-1^)		RE (%)	RR (mgl^-1^d^-1^)		RE (%)	RR (mgl^-1^d^-1^)	
	**25th day**	**10th day**	**25th day**	**25th day**	**10th day**	**25th day**	**25th day**	**10th day**	**25th day**
BOD	58.54	2.99	5.76	58.54	4.19	5.76	52.44	7.19	5.16
COD	81.25	96	41.60	87.50	32	44.80	62.50	44	32
Nitrate	89.52	13.19	8.94	88.91	12.50	8.88	84.72	18.26	8.46
Phosphate	87.83	348.35	688	79.02	1147.1	619	86.51	1555	677.68

*Values represent the mean ± SD of three replicates.*

#### COD

**FIGURE 3:**
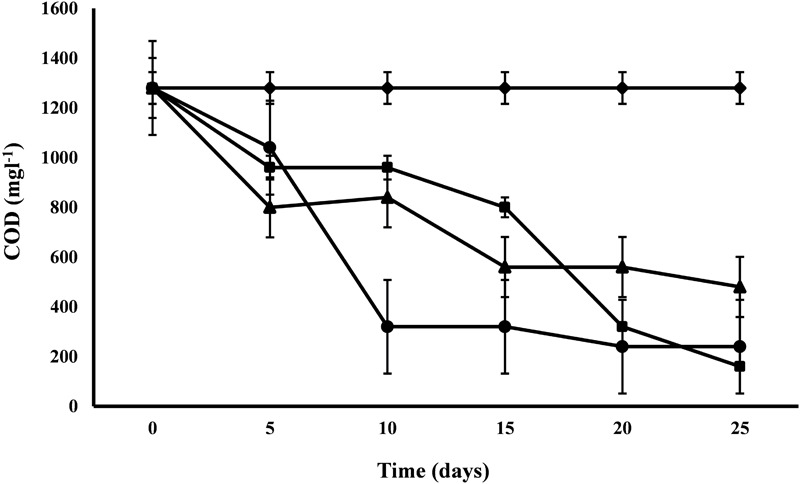
COD reduction at alternate of 5 days in 3:1 ratio of DWW treated with *A. ambigua, C. pyrenoidosa* and *S. abundans* (◆ -control; ■ -*C. pyrenoidosa*; ▲ -*S. abundans*; ● -*A. ambigua*) (Values represent the mean ± SD of three replicates).

COD exhibited a range of variations from 62.5 to 87.5% for the removal efficiency. The COD of control was 1280 mg l^-1^ and after 25 days of the experiment, *C. pyrenoidosa* removed maximum COD up to 160 mg l^-1^ followed by *A. ambigua* and *S. abundans* (Figure 3). Highest removal rate (96 mg L^-1^d^-1^) was exhibited by *S. abundans* and *A. ambigua* after 5th and 10th day of experiment respectively. *C. pyrenoidosa* showed a gradual reduction in the COD levels till the end of the experiment whereas *A. ambigua* showed a steep reduction till the 10th day. *S. abundans* did not show any notable reduction in the COD level after the 15th day of the experiment. COD is used as an indirect measure of the amount of organic compounds present in the wastewater and its reduction indicate that microalgae can have the inherent potential to utilize the organic compounds as an energy source besides carbon dioxide (Hu et al., 2012). High rate of COD removal is an indicator of the ability of microalgal species to tolerate and survive in the high COD levels (Wang et al., 2012).

#### Nitrate and Phosphate

**FIGURE 4:**
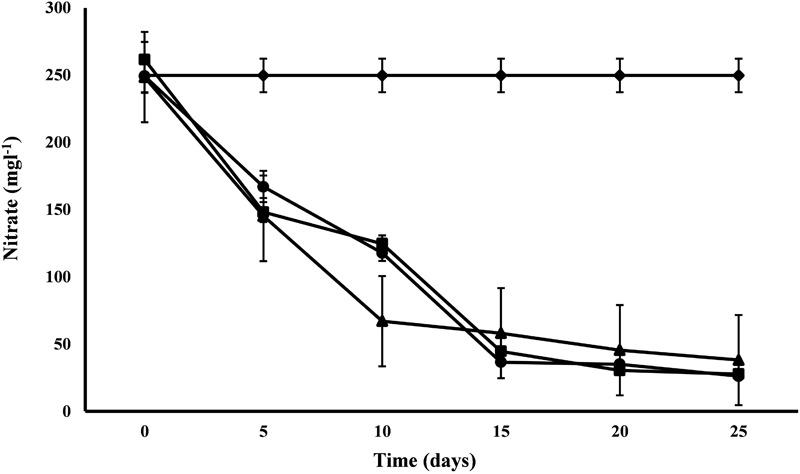
Nitrate reduction at alternate of 5 days in 3:1 ratio of DWW treated with *A. ambigua, C. pyrenoidosa* and *S. abundans* (◆ -control; ■ -*C. pyrenoidosa*; ▲ -*S. abundans*; ● -*A. ambigua*) (Values represent the mean ± SD of three replicates).

**FIGURE 5:**
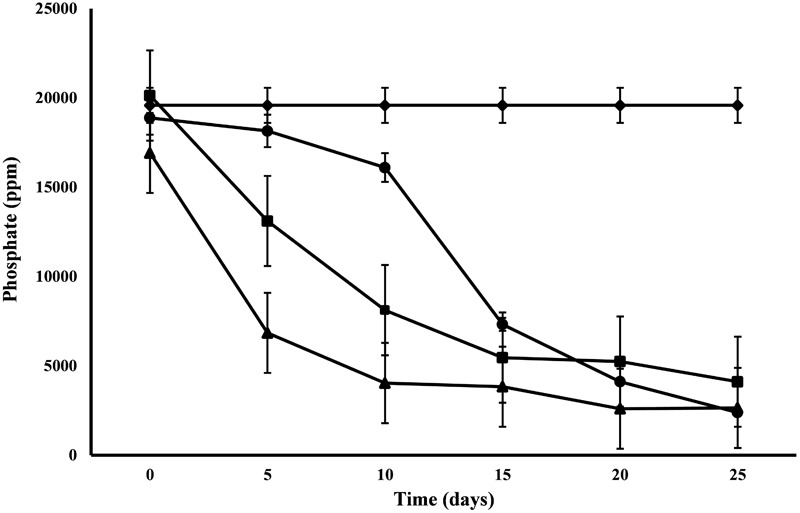
Phosphate reduction at alternate of 5 days in 3:1 ratio of DWW treated with *A. ambigua, C. pyrenoidosa* and *S. abundans* (◆ -control; ■ -*C. pyrenoidosa*; ▲ -*S. abundans*; ● -*A. ambigua*) (Values represent the mean ± SD of three replicates).

All the microalgal species used in the remediation experiment showed exceptionally good nitrate removal efficiency (85–88%). Highest removal rate was achieved by *C. pyrenoidosa* (13.67 mg L^-1^d^-1^) after the 15th day of experiment, closely followed by *A. ambigua* (13.19 mg l^-1^ d^-1^) after the 10th day. *A. ambigua* removed the maximum nitrate levels up to 26.15 mg l^-1^ after 25 days of remediation followed by *C. pyrenoidosa* (27.69 mg l^-1^) and *S. abundans* (38.12 mg l^-1^) whereas it was 249.66 mg l^-1^ in the control at the beginning of the experiment (Figure 4). The phosphate reduction efficiency in the 3:1 ratio of DWW was 87.83% for *A. ambigua*, 86.51% for *S. abundans* and 79.02% for *C. pyrenoidosa* (Table 3). Phosphate removal rate was highest for *S. abundans* followed by *A. ambigua* and *C. pyrenoidosa*. *A. ambigua* was observed to reduce the phosphate content at all the time intervals whereas *C. pyrenoidosa* and *S. abundans* showed an observable decrease till the 20th day of the experiment (Figure 5).

Cho et al. (2011) have reported the significant decrease in both the nitrate and phosphate concentrations (up to 92%) remediated by *C. vulgaris* grown in the municipal wastewater. The mechanism of phosphorous removal is more complex in comparison to nitrate and it is usually utilized in the form of orthophosphate for the assimilation by microorganisms. It is not only used for the growth and nucleic acid synthesis but also for the synthesis of value-added compounds (i.e., astaxanthin and polyunsaturated fatty acids) (Chen and Chen, 2006). Similarly, 96.8–98.4% decrease in ammonical nitrogen and 94.2–97.8% reduction in nitrate nitrogen was recorded by *Chlorella* sp. cultivated in the municipal wastewater (Kiran et al., 2014). Choudhary et al. (2016) have reported 87% COD and 70% nitrate removal in the 12 days experiment performed in the rural sector wastewater and observed that the algal growth induced a progressive decrease in the COD and nutrient values to levels that are lower than the permitted discharge limits. Wang et al. (2010a) assessed the phycoremediation potential of *Chlorella* sp cultivated for 21 days in the diluted digested manure (at various concentrations) and observed 76–83% reduction in the total nitrogen and 63–75% reduction in the total phosphorous content.

### Biochemical Characterization of Algal Biomass

The biochemical characterization of algal biomass will help to determine their industrial applications and their market value. If it contains high lipid and protein content, then can be used as oil and can decrease the market value of oil crops or if it contains high carbohydrate content, then it can reduce the market value of cereal crops (Tibbetts et al., 2015). Carbohydrates assist as structural components in the cell walls and storage components inside the cells (Ji et al., 2015). Total carbohydrate content in the oven dried algal biomass was measured through a colorimetric phenol sulfuric acid method (Table 4) and the maximum carbohydrate content (2363.75 ± 185.62 mg m l^-1^) was detected in the biomass produced from the *C. pyrenoidosa* over *S. abundans* and *A. ambigua* when grown in 3:1 ratio of DWW and *C. pyrenoidosa* showed significantly higher total carbohydrate content over the control.

**Table 4:** Biochemical characterization of algal biomass.

	*C. pyrenoidosa*		*A. ambigua*		*S. abundans*	
Parameters	Control	3:1 DWW	Control	3:1 DWW	Control	3:1 DWW
Crude protein (%)	50.17 ± 0.15	21.80 ± 10.76	52.16 ± 3.55	17.73 ± 6.24	30.63 ± 0.71	34.06 ± 2.09
Total Kjeldahl Nitrogen (mgl^-1^)	134.35 ± 5.61	519.40 ± 15.47	769.29 ± 19.01	260.27 ± 7.98	159.22 ± 4.55	350.23 ± 4.82
Total Carbohydrate (mg ml^-1^)	1790 ± 159	2364 ± 186	2270 ± 269	2193 ± 44	1804 ± 97	1114 ± 122
Lipid (μg ml^-1^)	42.01 ± 1.37	41.18 ± 0.58	43.15 ± 0.73	43.13 ± 0.58	41.08 ± 0.34	42.67 ± 2.41
Theoretical methane potential (ml CH_4_g^-1^ VS)	190.41 ± 4.85	99.79 ± 9.79	149.13 ± 6.67	29.82 ± 3.62	175.63 ± 14.79	54.46 ± 26.38

*Values represent the mean ± SD of three replicates with (p < 0.0001).*

**FIGURE 6:**
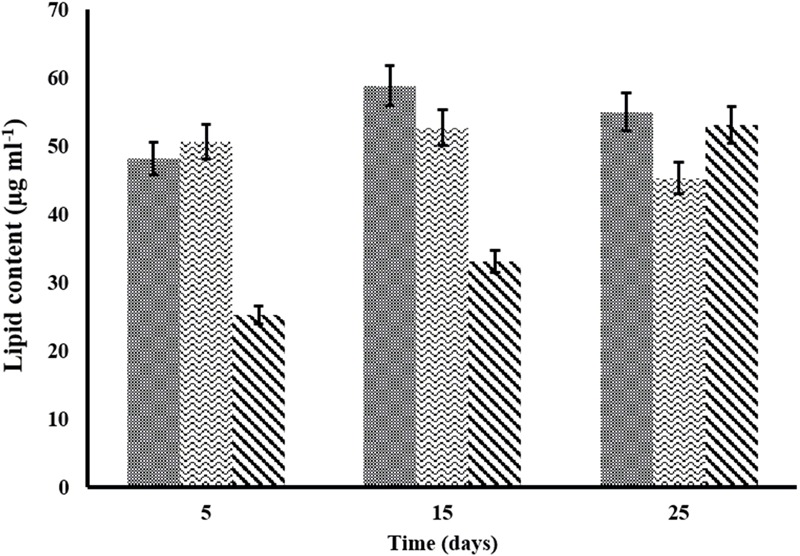
Variation in the lipid content of microalgal species at different time intervals in the 3:1 ratio of DWW (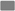 -*C. pyrenoidosa* 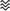 -*S. abundans* and 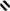 -*A. ambigua*). Values represent the mean ± SD of three replicates.

Lipid quantification is one of the important aspects of algal biomass characterization. Algal lipids comprise fatty acids and their constituent derivatives. Lipid content of *C. pyrenoidosa, A. ambigua* and *S. abundans* grown in the 3:1 ratio of DWW at different time intervals is shown in Figure 6. *C. pyrenoidosa* and *S. abundans* have shown maximum lipid content of 58.82 and 52.67 μg m l^-1^ respectively after 15 days of growth whereas, *A. ambigua* has shown maximum lipid content of 53.09 μg m l^-1^ after 25 days of growth (Figure 6). The lipid content was also determined in the oven dried algal biomass that was harvested after 25 days of the experiment (in the 3:1 DWW) at 60°C (Table 4) and was compared with the lipid content observed when the cultures were grown in the BG-11 medium alone. *S. abundans* showed slight increase in the lipid content grown in the 3:1 ratio of DWW (42.67 ± 2.4 μg m l^-1^) over the control (41.08 ± 0.34 μg m l^-1^) whereas *C. pyrenoidosa* showed slightly lower lipid content and *A. ambigua* was at par with the control in terms of total lipid content (Table 4). The lipid content was highest for *S. abundans* with 16.93% when grown in the 3:1 ratio of DWW over control and *C. pyrenoidosa* (10.36%) and *A. ambigua* (13.13%).

Malla et al. (2015) have reported the total lipid content of 20.69 and 28.32% in the Indian agricultural research institute and common effluent treatment plant wastewater respectively, based on the dry biomass of *C. minutissima*. Woertz et al. (2009) reported the total lipid content of biomass from the 10% dilution of DWW with tap water ranged from 8 to 14% and for 25% dilution ranged from 10 to 20% and postulated that nutrient stress leads to increase in the lipid content of algal biomass. Mahapatra et al. (2014) stated that the cultivation of microalgae in the carbon rich wastewater could result in the carbon accumulation of more lipids through the phenomenon of heterotrophy in which inorganic and organic forms of the carbon get converted into lipids.

The protein content was calculated by converting the elemental nitrogen content of the samples (dried algal biomass) measured with a CHNS-analyzer. *S. abundans* showed higher crude protein percentage in the biomass harvested from 3:1 ratio of DWW (34.06) over the control (30.63) whereas crude protein percentage was higher for control in other two algal species (Table 4). Among the three algal cultures, *S. abundans* showed higher protein content in the 3:1 ratio of DWW whereas *A. ambigua* showed the maximum protein content in control (BG-11 medium alone). *C. pyrenoidosa* and *S. abundans* have higher values of total Kjeldahl nitrogen when cultivated in the 3:1 ratio of DWW over the control (Table 4). Carbohydrate and lipid synthesis is a parallel phenomenon in the microalgae and the energy is first stored as carbohydrates and then excess energy is converted into lipids (Fan et al., 2014). The protein content for *C. pyrenoidosa* and *A. ambigua* was lower in 3:1 DWW over the BG-11 medium. It could be attributed to the presence of suspended particulate matter in the DWW that is not metabolized by the species and remained in the slurry (Wang et al., 2018).

### Theoretical Methane Potential

The elemental composition of dried algal biomass was used for the calculation of theoretical methane potential by Boyle’s equation. The calculated values of theoretical methane potential are mentioned in Table 4. Maximum theoretical potential was exhibited by the *C. pyrenoidosa* in the 3:1 ratio of DWW (190.41 ml CH_4_ g^-1^ VS) that was 1.9-fold lower in comparison to the control (BG-11 medium alone). *A. ambigua* and *S. abundans* showed only 20 and 31% methane potential respectively, when grown in the 3:1 ratio of DWW as compared to the methane potential of their control (BG-11 medium). The elemental analysis of the biomass revealed that *C. pyrenoidosa* and *A. ambigua* cultivated in the 3:1 DWW have higher C/N ratio of 12.01 and 11.12 respectively, as compared to the control (BG-11 media) (5.15 and 5.73 respectively). Statistical analysis (ANOVA) revealed that no significant interaction was observed between the microalgal species for lipid, carbohydrate, protein content and theoretical methane potential. However, the significant results were obtained for the interaction between control and 3:1 DWW (*p* < 0.0001) for each microalgal species.

The theoretical methane potential presents a crude estimate for the selection of microalgal species based on the balanced composition of carbohydrates, lipids and proteins for the successful production of fermentative biomethane. It helps to find the variations in the algal biomass due to their cultivation in the varying composition of wastewater that may affect their biochemical composition and the resultant methane potential (Choudhary et al., 2015). The application of such theoretical approach has been successfully applied for the estimation of methane potential of various solid wastes like sludge, leather fleshing and municipal solid waste and are useful for the evaluation of bioenergy and biomass potential of solid wastes for anaerobic digestion (Shanmugam and Horan, 2009).

## Conclusion

The study was conducted to investigate the potential of microalgal species for the remediation and bioenergy production when cultivated in the DWW. The current findings and the theoretical calculations have confirmed the coupling of wastewater remediation and bioenergy production. As evident from the results that the cultivation of microalgae in apt wastewater having the required concentrations of nitrogen and phosphorous can decrease the cost of biomass production by minimizing the external nutrient requirements. The results have supported the coupling of wastewater remediation and bioenergy production for the simultaneous management of DWW along with the generation of considerable amounts of renewable energy and decrease in secondary pollution. Furthermore, for the development of an effective cultivation and remediation process, optimization of certain parameters viz. pH, light, temperature, nutrients is required. Scaling–up of the derived process will further present an economic, eco-friendly solution to waste management.

## Data Availability

All datasets generated for this study are included in the manuscript and/or the supplementary files.

## Author Contributions

AB performed the experiments and wrote the manuscript. MK assisted in experiments and writing. NP conceptualized, supervised, wrote, and edited the manuscript.

## Conflict of Interest Statement

The authors declare that the research was conducted in the absence of any commercial or financial relationships that could be construed as a potential conflict of interest.
